# Genomic analysis of multidrug-resistant clinical *Enterococcus faecalis* isolates for antimicrobial resistance genes and virulence factors from the western region of Saudi Arabia

**DOI:** 10.1186/s13756-019-0508-4

**Published:** 2019-03-25

**Authors:** Muhammad Farman, Muhammad Yasir, Rashad R. Al-Hindi, Suha A. Farraj, Asif A. Jiman-Fatani, Maha Alawi, Esam I. Azhar

**Affiliations:** 1 Special Infectious Agents Unit, King Fahd Medical Research Center, King Abdulaziz University, Jeddah, 21589 Saudi Arabia; 2 Biology Department, Faculty of Science, King Abdulaziz University, Jeddah, 21589 Saudi Arabia; 3 Department of Medical Microbiology and Parasitology, Faculty of Medicine, King Abdulaziz University, Jeddah, Saudi Arabia; 4 Clinical and Molecular Microbiology Laboratories, King Abdulaziz University Hospital, Jeddah, Saudi Arabia; 5 Infection Control & Environmental Health Unit, King Abdulaziz University Hospital, King Abdulaziz University, Jeddah, Saudi Arabia; 6 Department of Medical Laboratory Technology, Faculty of Applied Medical Sciences, King Abdulaziz University, Jeddah, Saudi Arabia

**Keywords:** Multidrug-resistance, *Enterococcus faecalis*, Antimicrobial resistance genes, Multilocus sequence typing, Saudi Arabia

## Abstract

**Background:**

*Enterococcus faecalis* is a ubiquitous member of the gut microbiota and has emerged as a life- threatening multidrug-resistant (MDR) nosocomial pathogen. The aim of this study was to survey the prevalence of multidrug-resistant and epidemiologically important strains of *E. faecalis* in the western region of Saudi Arabia using phenotypic and whole genome sequencing approaches.

**Methods:**

In total, 155 patients positive for *E. faecalis* infection were included in this study. The isolates were identified by MALDI-TOF, and screen for antimicrobial resistance using VITEK-2 system. Genome sequencing was performed with paired-end strategy using MiSeq platform.

**Results:**

Seventeen sequence types (STs) were identified through multilocus sequence typing (MLST) analysis of the *E. faecalis* genomes, including two novels STs (ST862 and ST863). The most common STs in the Saudi patients were ST179 and ST16 from clonal complex 16 (CC16). Around 96% (*n* = 149) isolates were MDR. The antibiotics quinupristin/dalfopristin, clindamycin, and erythromycin demonstrated almost no coverage, and high-level streptomycin, gentamycin, and ciprofloxacin demonstrated suboptimal coverage. Low resistance was observed against vancomycin, linezolid, and ampicillin. Moreover, 34 antimicrobial resistance genes and variants, and three families of insertion sequences were found in the *E. faecalis* genomes, which likely contributed to the observed antimicrobial resistance. Twenty-two virulence factors, which were mainly associated with biofilm formation, endocarditis, cell adherence, and colonization, were detected in the isolates.

**Conclusions:**

Diverse STs of *E. faecalis*, including strains associated with common nosocomial infections are circulating in the healthcare facility of Saudi Arabia and carried multi-drug resistance, which has important implications for infection control.

**Electronic supplementary material:**

The online version of this article (10.1186/s13756-019-0508-4) contains supplementary material, which is available to authorized users.

## Background

Over the past decades, enterococci have emerged as the most prevalent nosocomial pathogens. Once viewed as part of the normal gut flora with little clinical significance, and they are now recognized as the cause of several types of community- and hospital-acquired infections, including life-threating bloodstream infections, endocarditis, meningitis, and urinary tract infections [1]. Disease outbreaks from antimicrobial-resistant enterococci have occurred in the United States and Europe [2, 3], and the European Centre for Disease Prevention and Control (ECDC) has reported that enterococci are the most commonly isolated bacteria after *Escherichia coli* and *Staphylococcus aureus* from healthcare-associated infections in Europe [4]. Furthermore, nosocomial infections caused by vancomycin-resistant enterococci (VRE) represent a serious clinical problem in healthcare facilities in many countries worldwide [5, 6].

*Enterococcus faecalis* is the most common enterococcal species associated with nosocomial infections, accounting for 80–90% of the infections, followed by *Enterococcus faecium* (5–10%) [7]. Other species of enterococci rarely cause infection [2]. Genotyping of *E. faecalis* and *E. faecium* by multilocus sequence typing (MLST) and other methods revealed that distinct clones of these species are associated with hospital infections/outbreaks; these clones are referred to as high-risk enterococcal clonal complexes [8, 9]. In recent years, these species have shown increasing resistance to several antibiotics, including penicillin, aminoglycosides, and glycopeptides, which thus limits antimicrobial therapeutic options [10]. Glycopeptide resistance may be due to the acquisition of *van* genes, whereas *vanC*1 and *vanC*2/3 are responsible for intrinsic resistance in enterococci [11]. Vancomycin resistance that mainly arises from the *van*A gene cluster is commonly identified on the mobile genetic element Tn1546 [12]. The mobile genetic elements from enterococci have also recently been shown to be able to transfer vancomycin resistance to more pathogenic bacteria such as *S. aureus* [13]. Monitoring the antimicrobial resistance (AMR) in enterococci from clinical specimens is essential for controlling the spread of resistance genes against vancomycin and other antibiotics.

Available literature has highlighted an increasing prevalence of multi-drug resistant (MDR) *E. faecalis* in the eastern region of Saudi Arabia [14]. However, no molecular characterization of MDR *E. faecalis* isolates from Saudi Arabia, and neighboring countries has been performed. An active molecular epidemiology program is critical for creating basic knowledge about local microorganisms and their resistance to refine policies on controlling infections from antimicrobial-resistant bacteria in hospitals and other healthcare facilities within the country. The main objective of this study was to perform antibiotic susceptibility, and genomic analysis of clinical *E. faecalis* isolates from the western part of the country. The isolates were evaluated for the presence of virulence and antimicrobial resistance genes (ARGs). MLST analysis was performed to track the global distribution of the *E. faecalis* sequence types (STs) identified in this study.

## Materials and methods

### Samples collection

King Abdulaziz University Hospital (KAUH) is an 845-bed teaching hospital that mainly serves the western region of Saudi Arabia. The *E. faecalis* strains isolated from patients at KAUH in 2014–2015 were included in this study. Demographic information and medical history were obtained from patients’ electronic medical records, which are prospectively maintained. This study was reviewed and approved by the ethical research committee of the Faculty of Medicine at King Abdulaziz University under the reference number (235–15). In total, 155 nonduplicate clinical isolates of *E. faecalis* were analyzed in this study.

### Identification and antimicrobial susceptibility screening

The purified isolates were freshly cultured on Columbia blood agar plates at 37 °C for 20 h using a biosafety level-2 cabinet and stored at − 80 °C in 15% glycerol and 1% skim milk. Isolates were identified by high-throughput MALDI-TOF using a VITEK-MS (bioMérieux, France) system following the manufacturer’s protocol [15]. The calibration was performed using standard *Escherichia coli* ATCC 25922 to validate the run. All isolates were tested for antimicrobial susceptibility using an automated VITEK-2 (bioMérieux) system with a specific AST-GP2 card. VITEK 2 system used broth microdilution minimum inhibitory concentration method for susceptibility testing and perform repetitive turbidimetric monitoring of bacterial growth during an abbreviated incubation period. MIC results were interpreted based on the Clinical and Laboratory Standards Institute guidelines [16]. The criteria of Magiorakos et al. was used to defined MDR isolates [17].

### Amplification of the AMR genes

Genes responsible for AMR to vancomycin were amplified from the phenotypically resistant *E. faecalis* isolates using primers, and PCR conditions described previously [18]. Gel-purified amplified PCR products were sequenced with ABI prism sequencer 3730 (Applied Biosystems, USA). NCBI nucleotide BLAST was used to confirm the amplification of the respective genes.

### PFGE and whole genome sequencing

Genotyping of the *E. faecalis* isolates was performed using PFGE in a CHEF-DR II apparatus (Bio-Rad, USA) as previously described [19], with some modifications. The optical density of an *E. faecalis* suspension was adjusted to 1 at 600 nm. The *Sma*I (60 units) restriction enzyme (Thermo Fisher Scientific, USA) was used for digestion of the plugs. PFGE was performed for 24 h at 6 V, with an initial switch of 3.5 s and the final switch at 23.5 s. *Sma*I-digested *S. aureus* strain NCTC8325 was used as a DNA molecular size control. Forty-four representative isolates from PFGE bands patterns were selected for whole genome sequencing. Genomic DNA was extracted from *E. faecalis* isolates using UltraClean® Microbial DNA isolation kit (MO BIO Laboratories, Inc. USA). Genomic libraries were prepared using Nextera XT DNA library preparation kit (Illumina, Inc., USA) and sequencing was performed using V3, 2 × 300 bp chemistry on a MiSeq platform (Illumina, Inc., USA).

### Data analysis

BioNumerics software (V 7.6.0) from Applied Maths was used to analyze the bands patterns from PFGE, and a dendrogram was constructed using unweighted pair group with arithmetic mean (UPGMA). The default parameters of a 1% tolerance and an 85% similarity index were used for clustering the isolates.

The generated reads from whole genome sequencing were filtered according to the read qualities. The genome assemblies were prepared with SPAdes 3.9 algorithm, and sequence reads were mapped to clinical isolate *E. faecalis* reference genome V583. Single nucleotide polymorphisms (SNPs) were determined in the core genomes and were used to construct a maximum likelihood phylogenetic tree using CSI Phylogeny 1.4 [20]. The phylogenetic tree was visualized using Interactive Tree of Life (iTOL) tool [21]. ARGs were identified using ResFinder3.1 [22], ARG-ANNOT (Antibiotic Resistance Gene-ANNOTation) [23], and CARD (Comprehensive Antibiotic Resistance Database) [24]. VirulenceFinder 2.0 was used to identify virulence-associated genes [25]. Insertion sequences were identified using ISfinder [26, 27] and were reconfirmed from the BLASTn at NCBI. To track the epidemiology in a global context, the sequence types (STs) of the isolates were identified using an MLST scheme based on seven housekeeping genes [9]. The similarities between different STs were investigated using BioNumerics (V 7.6.0) with UPGMA and categorical coefficient of similarity. STs were grouped into clonal complexes based on single and double locus variants using eBburst (http://eburst.mlst.net). Pearson chi-square or likelihood ratio was used with a threshold of *p* < 0.05 for statistical significance. SPSS version 22 was used for statistical analysis. Genome sequences were deposited into the European Nucleotide Archive (ENA) under accession number ERS2489758-ERS2489715.

## Results

### Demographic and clinical characteristics

The majority of the isolates were recovered from inpatients (*n* = 102, 65.8%). In 75.5% of cases, infection was community acquired, and in 24.5% of cases, infection was probably hospital acquired. In the latter cases, infection was detected in patients after 72 h of admission in the hospital (Additional file 1: Table S1). Isolates were recovered from patients with various diseases, which were broadly classified as malignancy, renal and kidney-associated disease, urinary tract infection (UTI), gastrointestinal tract infection, respiratory tract infection, and pregnancy (Additional file 1: Figure S1). Healthcare-associated infection was detected in eight patients with a malignancy. Community-acquired infection with *E. faecalis* was most frequently observed in patients with chronic kidney disease and renal failure (*n* = 22, 14.2%) or UTI (*n* = 13, 8.4%) and in pregnant patients (n = 13, 8.4%) (Additional file 1: Figure S1). The majority of the isolates were recovered from patients who were > 50 years old (*n* = 67, 43.2%). Importantly, 23 (14.8%) of the *E. faecalis* isolates were recovered from neonates (≤1 year), and 19 of whom were inpatients (Additional file 1: Table S1). The isolates were recovered from heterogeneous clinical specimens, mostly urine-midstream (*n* = 58, 37.4%) and urine-catheter (*n* = 49, 31.6%) followed by wound swab (*n* = 13, 8.4%) and blood (*n* = 11, 7.1%). The strains were mainly isolated from Saudi patients (*n* = 81, 52.3%) and expatriates representing 16 different nationalities (Additional file 1: Figure S2). Other demographic information is presented in Additional file 1: Table S1.

### Antimicrobial susceptibility analysis

**Fig. 1:**
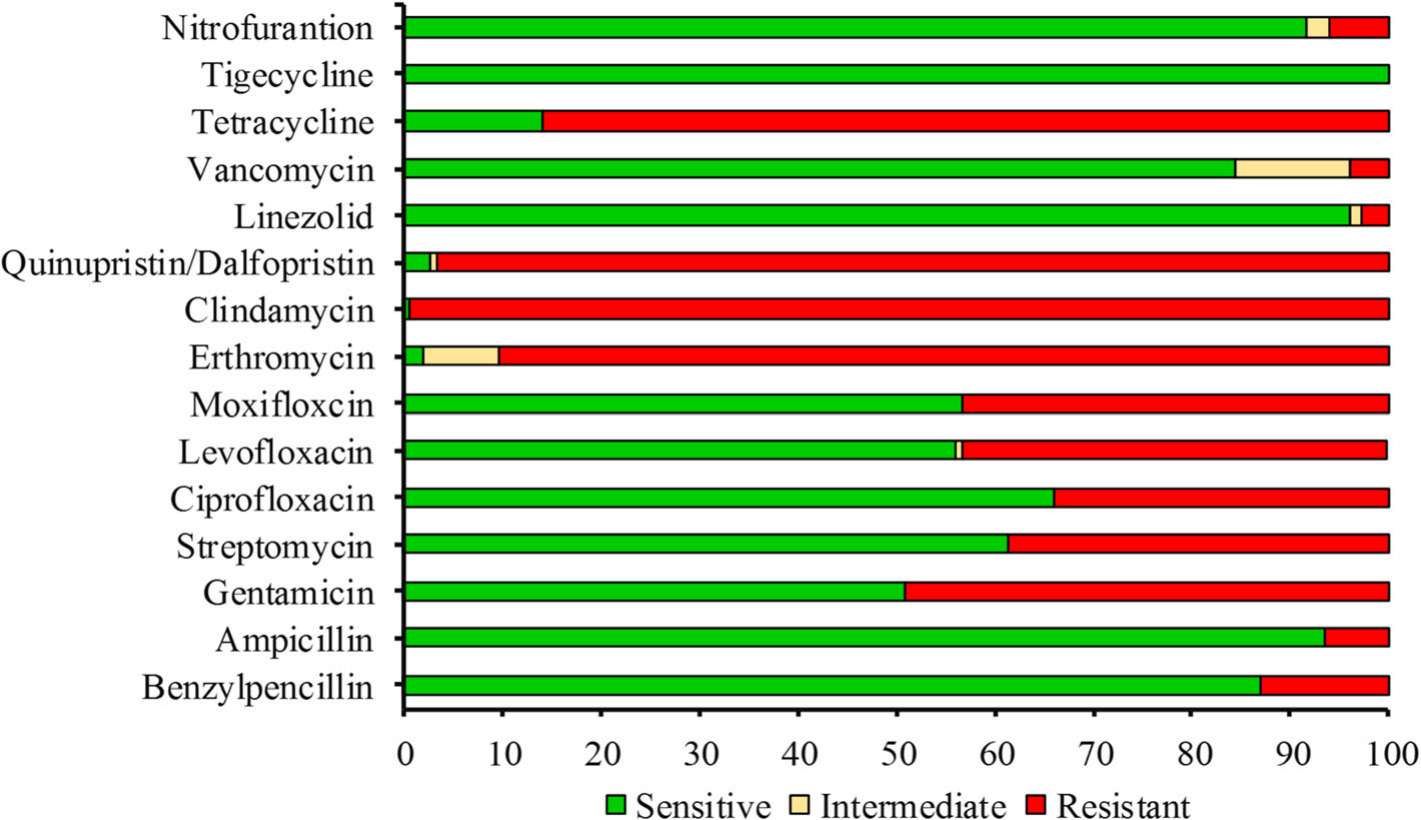
Antimicrobial resistance and susceptibility profile of the tested antibiotics against *E. faecalis* isolates. The x-axis values are expressed in percentage

The majority of the *E. faecalis* isolates (96.1%) had an MDR pattern. In total, 60% of the isolates were resistant to ≥5 tested antibiotics, and eight isolates were resistant to ≥10 antibiotics from different classes and mainly were from macrolide, quinolone, tetracycline, lincosamide, and streptogramin. The isolates Efs236 and Efs249, recovered from wound swab and urine-midstream, were found to be resistant to 11 and 12 antibiotics, respectively. Ampicillin resistance was detected in 10 isolates with a MIC range of 8–32 μg/ml. Six of the isolates were resistant to vancomycin (MIC ≥32 μg/ml). The highest resistance was observed against clindamycin (99.3%). More than 85% of the isolates were resistant or intermediately resistant to erythromycin, quinupristin/dalfopristin, and tetracycline (Fig. 1). Forty-nine percent of the isolates were resistant to high-dose gentamycin, and 38.7% were resistant to high-dose streptomycin (Fig. 1). For the quinolone group, 43.2% isolates were resistant to moxifloxacin and levofloxacin, whereas 34.2% isolates were resistant or intermediately resistant to ciprofloxacin. Four of the isolates were resistant to linezolid. All the isolates were susceptible to tigecycline (Fig. 1).

### Evaluation of AMR pattern

In total, 37 different patterns of antibiotic resistance were recorded for 155 isolates, including 13 groups and 24 unique patterns (Additional file 1: Figure S3). The most dominant pattern (P1) of resistance involved four antibiotics (tetracycline, erythromycin, clindamycin, and quinupristin/dalfopristin) and was observed in 38 isolates, followed by the P2 pattern of resistance to nine antibiotics in 26 isolates. Antibiotype pattern P3 was identified in 18 isolates that harbored resistance to five antibiotics. Antibiotype pattern P13 was detected in two isolates that were resistant to 12 antibiotics (Additional file 1: Figure S3).

### PCR analysis of vancomycin resistance genes

Phenotypically vancomycin resistant isolates were further characterized for the respective *van* genes. Vancomycin-resistant isolates mainly carried the *van*A gene (5/6), and one isolate was positive for *van*B; *van*C1/C2 was not detected in any of the vancomycin-resistant isolates. The Efs251 isolate showed PCR amplification of the *van*A gene cluster.

### PFGE and genome analysis

**Fig. 2:**
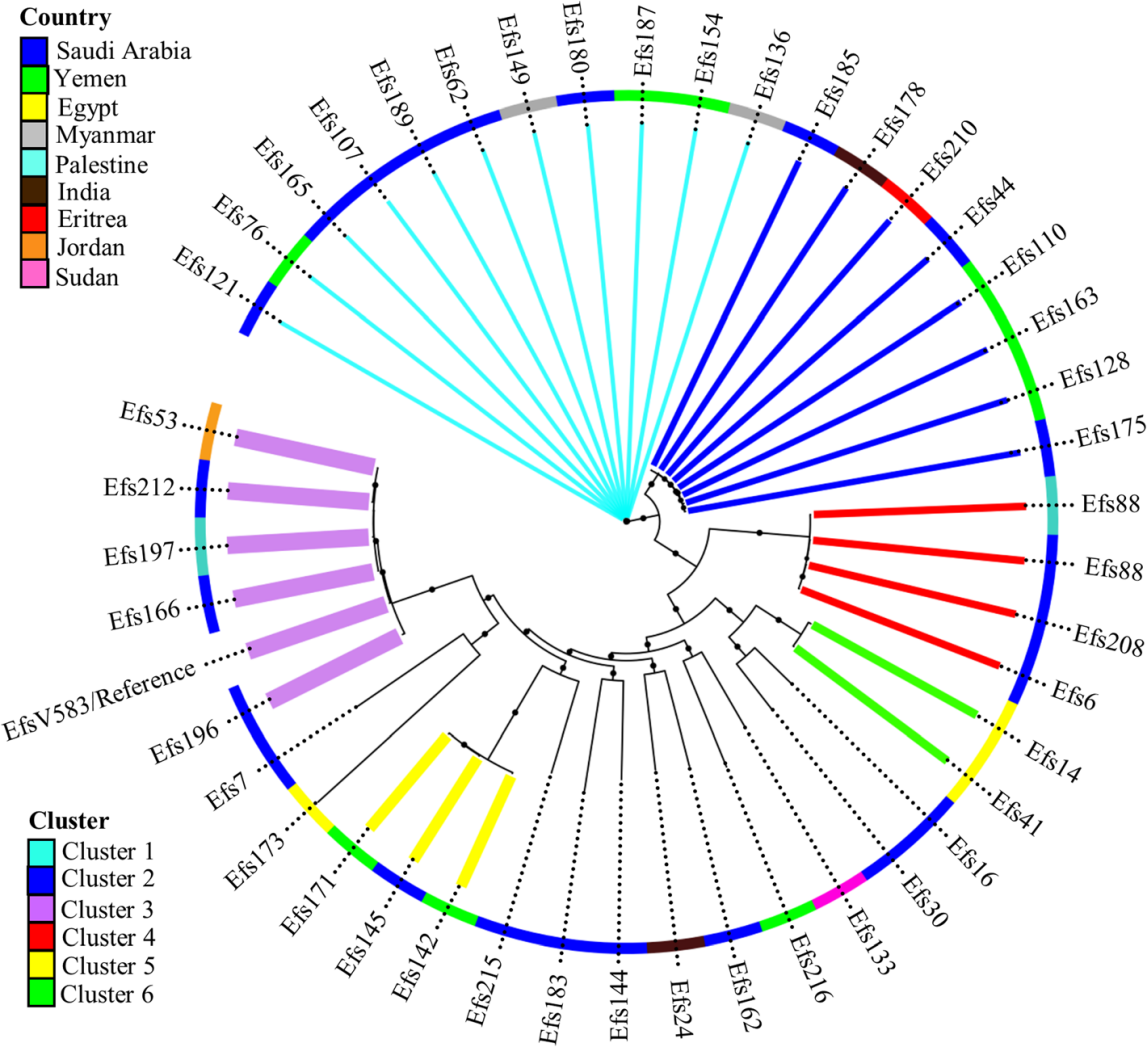
Phylogenetic linkage among *E. faecalis* isolates based on SNPs in core genomes. Colored branches indicate the three dominant lineages and the outer colored ring indicates the nationality

PFGE revealed substantial heterogeneity in the *E. faecalis* isolates. The isolates were clustered into 44 clades at 85% similarity index in the UPGMA dendrogram constructed from the bands patterns (Additional file 1: Figure S4). A representative isolate from each clade underwent genome sequencing. Genomes of the 44 isolates covered 70.6% of the genome of the reference strain V583. The individual isolates covered the reference genome within a range of 2,501,623 to 2,987,577 nucleotides. A phylogenetic tree based on SNPs grouped 44 isolates into three clusters and two minor clusters (Fig. 2). Annotation of the tree with geographical region indicated clusters 1, 3, 4, and 5 were mainly found in the patients from the Middle East, whereas cluster 2 isolates were found in patients of different nationalities (Fig. 2).

### MLST analysis and identification of clonal complex

**Fig. 3:**
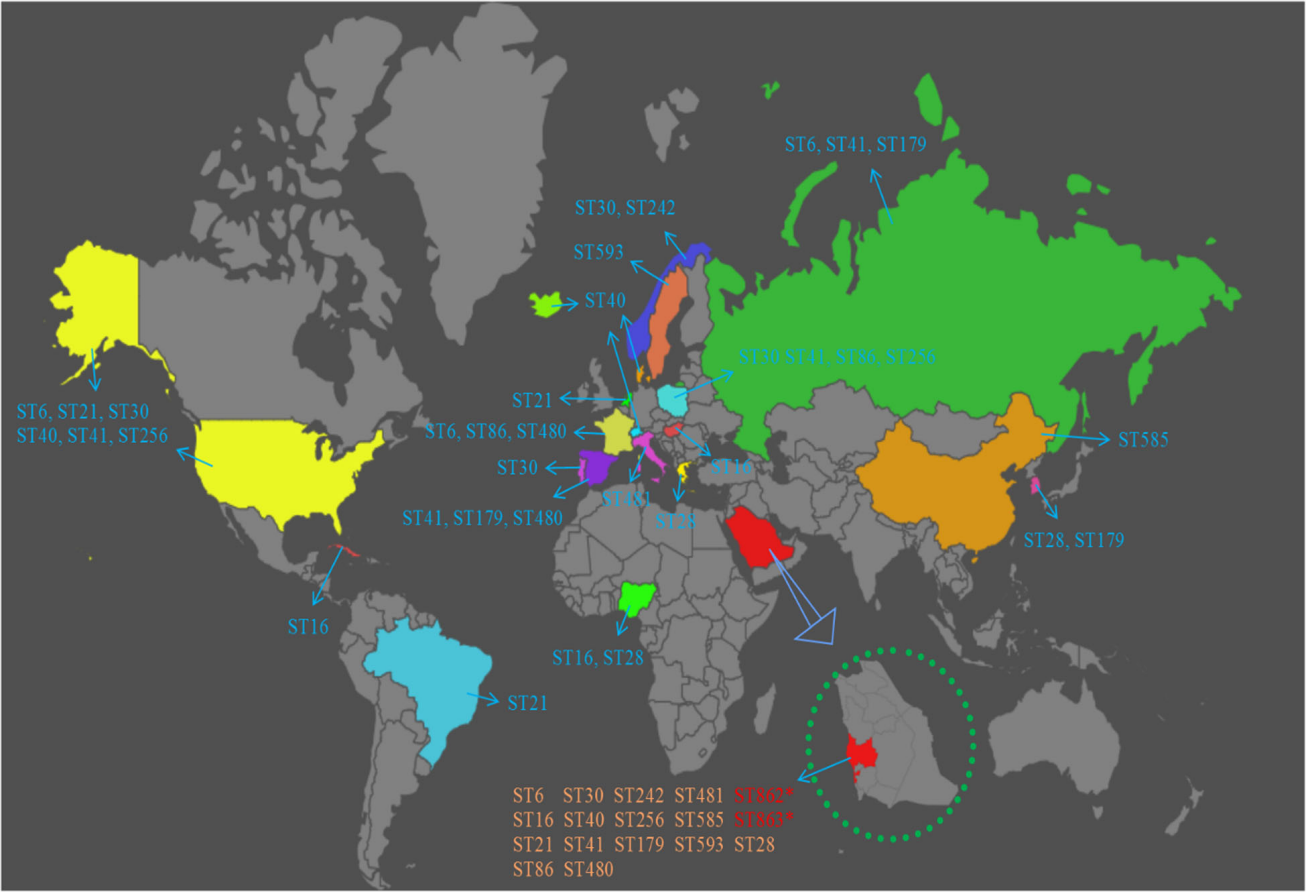
Global distribution of *E. faecalis* STs identified in Saudi Arabia. New STs are presented in red color, whereas other STs were found in different countries

**Fig. 4:**
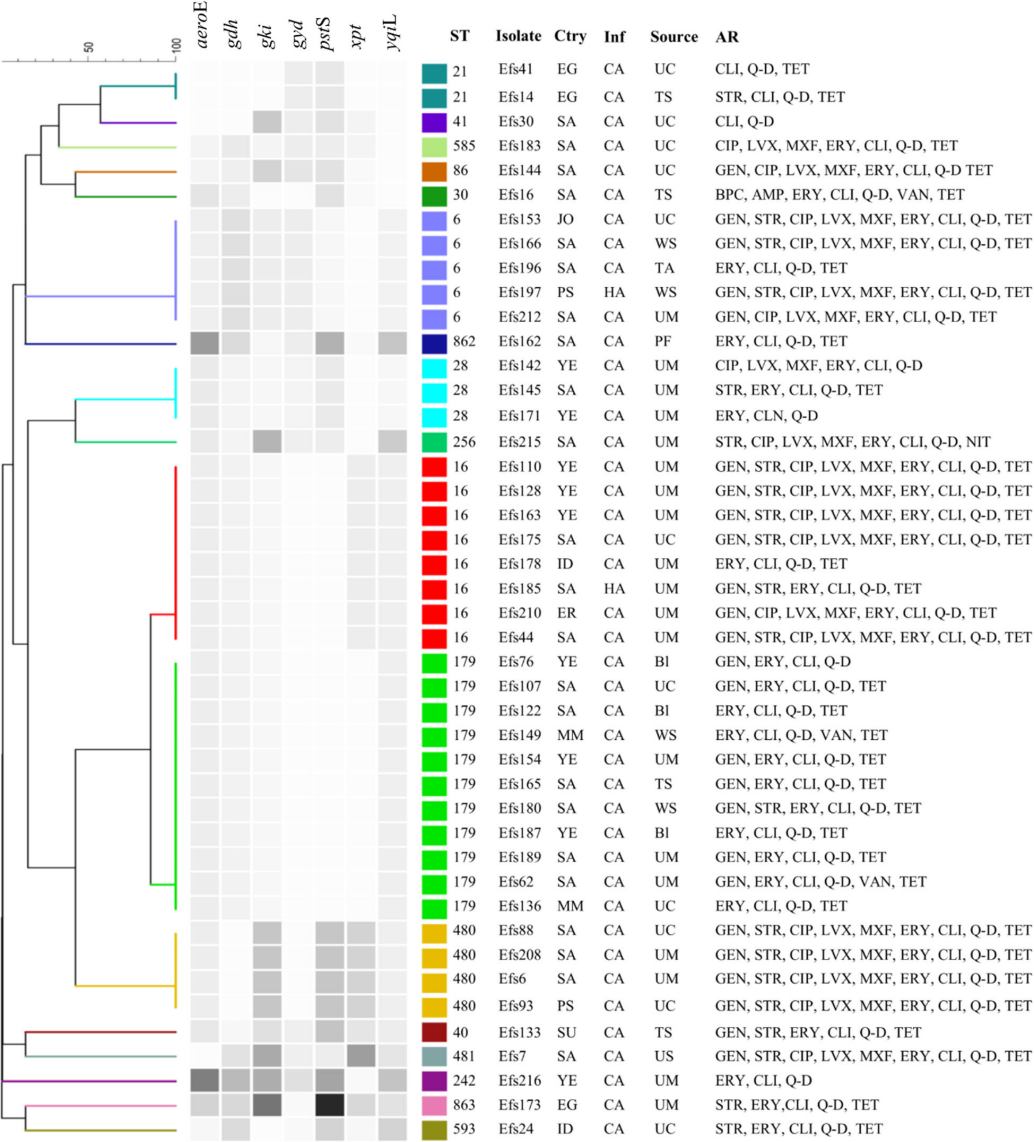
UPGMA dendrogram from the pattern of pairwise differences in alleles that revealed the genetic relationships of STs among the *E. faecalis* isolates, along with the country (Ctry), infection (Inf), specimen source, and antibiotic resistance (AR) information. HA, hospital-acquired; CA, community-acquired; UC, urine-catheter; UM, urine-midstream; Bl, blood; WS, wound swab; TAS, tracheal aspirate; TS, tissue swab; PF, peritoneal fluid

We assigned sequence types (STs) to the genome sequences of the 44 isolates, which accounted for 17 STs, including two novels STs (ST862 and ST863) based on new combinations of the known alleles (Fig. 3). In addition, all 17 STs were reported for the first time from Saudi Arabia and showed global distribution (Fig. 3). Eleven isolates were grouped together under a single dominant sequence type ST179 (25%, *n* = 11), followed by ST16 (18.2%, *n* = 8). Other STs were identified in ≤5 isolates. Isolates from clusters 1 and 2 from SNPs tree were assigned to ST179 and ST16, respectively (Fig. 2). Clusters 3, 4, and 5 were assigned to ST28, ST480, and ST128, respectively. eBURST resolved 17 isolates with a unique allelic profile into one group and 15 singletons at single-locus and double-locus variants (Additional file 1: Figure S5). The blast with MLST database through eBURST grouped the identified STs into 14 distinct clonal complexes (CC) including major CC16, which comprised ST16 and single-locus variant ST179 followed by ST6 from CC2 (Additional file 1: Figure S5). In total, 12 different STs were detected in Saudi patients, including a novel ST862 recovered from pleural fluid (Fig. 4). The second novel ST863 was identified from urine-midstream of an Egyptian patient. Five STs were common between Saudi and expatriate populations (Fig. 4). ST16 and ST480 were recovered mainly from urine-midstream and urine-catheter samples, whereas other STs were recovered from heterogeneous clinical samples (Fig. 4). No significant association was observed between STs and diagnosed disease type; for example, ST6, ST16, and ST480 were detected in samples from multiple hospital units and patients with different diseases. However, the ST179 isolates were found at relatively higher numbers in the pediatric ward. All isolates of ST16 and ST179 from CC16 and ST6 from CC2 were resistant to high-dose gentamicin (Fig. 4). However, the ST179 isolates were susceptible to the tested antibiotics from the quinolone group (Fig. 4). A similar pattern of resistance against nine antibiotics was observed in the ST480 isolates. No specific prevalence of vancomycin-resistant isolates was noticed in any ST.

### ARGs and insertion sequence families

**Fig. 5:**
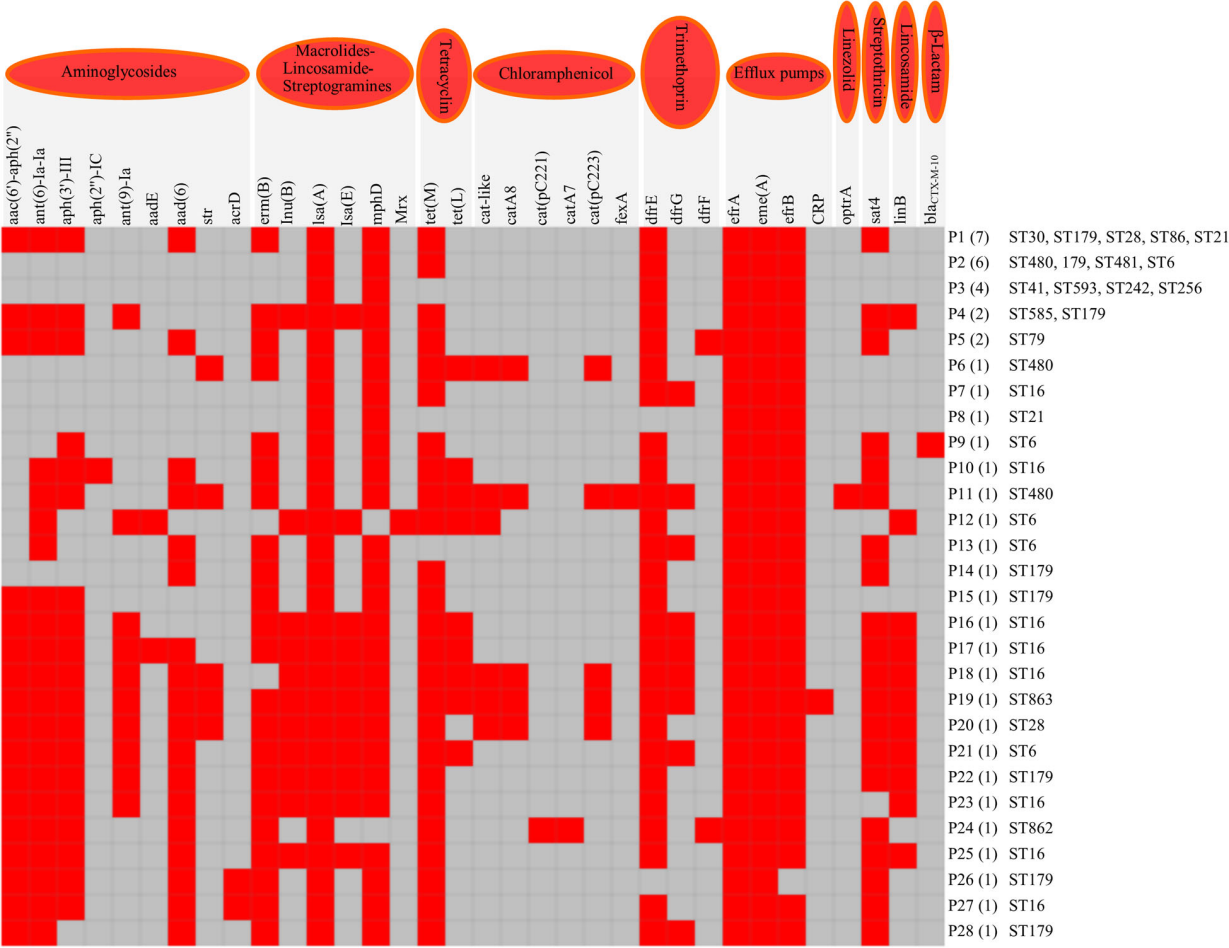
Antimicrobial resistance genes patterns identified from genome sequencing of 44 *E. faecalis* isolates. Total 34 resistance genes and variants were found in the analyzed genomes

In total, 34 ARGs and variants were detected in the genome-sequenced isolates. The frequency of ARGs was found to be in the range of 5–24 genes, whereas nine isolates predominantly carried 13 ARGs followed by six isolates that harbored seven ARGs (Fig. 5). A maximum of 24 ARGs were found in the isolate EFs93 and 22 ARGs in EFs208. Two other isolates had 20 ARGs (Fig. 5). Moreover, aminoglycoside, trimethoprim, and efflux pump-mediated resistance genes were found in most of the isolates (Additional file 1: Figure S6). Aminoglycoside resistance was conferred by the presence of nine resistance genes, while *aph*(3′)-III gene was found in 27 isolates and *aac*(6′)-*aph*(2″) and *aad*(6) were found in ≥25 isolates (Additional file 1: Figure S6). Streptomycin resistance was conferred by the most common genes, *ant*(6)-*Ia*, which was detected in 29 isolates. Macrolide and streptogramin-resistance gene *isa*A was found in all sequenced isolates, while *mph*D and *erm*(B) genes were found in 42 and 30 isolates, respectively (Additional file 1: Figure S6). Most of the isolates were carrying efflux-associated ARGs (*efr*A, *eme*A, and *efr*B). Among the three trimethoprim-resistance genes, *dfr*E and *dfr*G were detected in 42 and 9 genomes, respectively. Tetracycline-resistance *tet*(M) gene was found in 38 genomes (Additional file 1: Figure S6)*.* One isolate demonstrated horizontally acquired resistance gene *optr*A, which is responsible for linezolid resistance.

Three families of insertion sequences (ISs) IS*30* (IS*6770,* IS*1062*, and IS*Enfa364*), IS*256* (IS*Ef1* and IS*16*), and IS*1182* (IS*Enfa2*) were identified in the *E. faecalis* genomes. Distribution of the IS families and their members are summarized in Additional file 1: Table S2, illustrating their abundance and association with *E. faecalis* STs. Family IS*30* (*n* = 24) was found at a relatively higher frequency, followed by IS*256* (*n* = 16) and IS*1182* (*n* = 3).

### Virulence-associated genes analysis

A total of 22 putative virulence genes were identified, among which sex pheromone (*cOB*1, *cad*, and *cCF*10), Sortase (*srt*A), endocarditis and biofilm-associated pili genes (*ebp*B, *ebp*A, and *ebp*C), adhesin (*efaAfs*, and *ace*), thiol peroxidase (*tpx*), and *E. faecalis* virulence factor *Elr*A genes were commonly detected in ≥97.7% genomes from 44 isolates (Additional file 1: Figure S7). Hyalurodinase gene *hyl*A was identified in 38 genomes. Overall, 14–22 virulence-associated genes were detected in the analyzed genomes. Cytolysin toxin-associated genes (*cyl*A, *cyl*B, *cyl*L, and *cyl*M) were more prevalent among ST6, ST16, ST28, and ST179.

## Discussion

Consistent with the international trend, the prevalence of enterococcal infections is increasing in Saudi Arabia, and *E. faecalis* is one of the most common enterococcal species isolated from hospital-associated infections. The ECDC has estimated that enterococci are responsible for 8% of the healthcare-associated infections on average in Europe, and the agency has placed them in the category of pathogens posing a major threat to healthcare systems [4]. Enterococcal species are intrinsically resistant to a broad range of antibiotics, such as cephalosporins and sulfonamides [28]. They represent a major infection control challenge because of their ability to acquire additional resistance through the transfer of plasmids and transposons and because they can disseminate easily in the hospital environment.

In this study, linezolid, tigecycline, and vancomycin demonstrated > 95% activity against *E. faecalis* isolates in vitro. The antibiotics quinupristin/dalfopristin, clindamycin, and erythromycin demonstrated almost no coverage, and other antibiotics such as high-dose streptomycin, gentamycin, and ciprofloxacin demonstrated suboptimal coverage. Previous studies from Saudi Arabia revealed 21–25% resistance to high-dose gentamycin and 11–13% resistance to high-dose streptomycin [29, 30]. The high level of aminoglycoside resistance observed in this study is highly concerning, given that aminoglycosides are used in combination with other active molecules, mainly β-lactams, to treat enterococcal infections such as enterococcal endocarditis [28]. In particular, gentamycin is used as a synergistic antibiotic with ampicillin [31], and amoxicillin is the first choice of treatment for *E. faecalis* causing UTIs [32]. In contrast to a study from the eastern region of Saudi Arabia, comparatively lower resistance to ampicillin and higher resistance to erythromycin, gentamycin, streptomycin, and tetracycline were observed in this study, suggesting the diverse geographical distribution of MDR *E. faecalis* isolates in Saudi Arabia [30, 33].

The rate of vancomycin-resistant *E. faecalis* in this study was slightly higher than that found in a previous study from Saudi Arabia that was conducted at King Khalid Hospital in Riyadh. That study identified the vancomycin-resistant phenotype in 0–1.8% of isolates [30]. The resistance rates reported by the National Healthcare Safety Network during 2009–2010 was between 6.2% and 9.8% for *E. faecalis*, depending on the site of infection [34]. Despite the increasing number of reports of VRE in different geographical regions of the world, there is a distinct lack of data regarding the molecular characterization of VRE isolates originating from the Middle East region. No genotypic characterization of vancomycin-resistance genes and other acquired AMR genes in *E. faecalis* has been described in the available literature from Saudi Arabia. In this study, acquired *van*A and *van*B genes were identified in the vancomycin-resistant *E. faecalis* isolates from patients in the western region of Saudi Arabia. No intrinsic resistance genes (*van*C1/C2) were detected in the tested isolates. Similar results have been reported in Europe, where a mix of VRE carrying *van*A and *van*B were found [6]. *Enterococcus faecalis* isolates tested in this study also possessed *gyr*A, and *par*C, genes conferring resistance to quinolone groups of antibiotics including ciprofloxacin, levofloxacin, and moxifloxacin that are commonly prescribed for UTIs, enteric infections, and respiratory tract infections.

Consistent with previous studies, the most common aminoglycoside-modifying enzyme genes (*aac*(6′)-Ie-*aph*(2″)-Ia and *ant*(6′)-Ia) were found in the *E. faecalis* isolates that were resistant to high-dose gentamycin and streptomycin [35]. Two mechanisms are responsible for the cross resistance to macrolide-lincosamide-streptogramin A in *E. faecalis*, including an intrinsic *lsa* gene and a change in the target site of erythromycin that is mediated by the *erm*(B) gene [36, 37]. In this study, we found the *lsa* gene in all genome sequence isolates, and the *erm*(B) gene was found in 68.1% isolates, similar to previous studies from the United States, China, and Korea [36, 38, 39]. The results obtained in this study are consistent with a previous report indicating that 98% of the *E. faecalis* isolates possess *eme*A gene and ATP-binding cassettes (ABC), which are *efr*A and *efr*B [40].

The ability of enterococci to form biofilm contributes to the pathogenicity of the bacteria in nosocomial infections because mature biofilms of *E. faecalis* can withstand antimicrobial agents up to 100- to 1000-fold concentrations. In contrast to previous studies, a large group of virulence factors was found in the genome sequences of *E. faecalis* isolates, as seen in other Gram-positive cocci, such as β-hemolytic *Streptococcus* and *S. aureus*. In this study, 22 virulence genes were retrieved from the *E. faecalis* isolates, including the genes associated with biofilm formation as previously mentioned. The presence of virulence determinants in enterococcal isolates assist them in acquiring adaptive elements that provide them with evolutionary benefits for relative fitness in hospital settings [41, 42]. In comparative studies, *E. faecalis* is considered to be inherently more virulent and to have greater capability to acquire virulence factors than *E. faecium* strains [1]. Overall, variation was observed in the relative distribution of virulence factors in the enterococcal species from different geographical regions [43, 44]. Findings from the current study are consistent with a previous report that *esp* and *cyl*A genes were mainly found in CC16 isolates. Importantly, the cCF10 gene, which activates the conjugation of pCF10 plasmid, was found in this study. This plasmid plays an important role in the dissemination of virulence factors and resistance genes among enterococci [45].

A noticeable diversity of strains was observed in the *E. faecalis* isolates from Saudi Arabia, and 17 distinct STs, including two novel STs, from the genome sequences of 44 isolates were identified. Major clusters from a phylogenetic tree based on SNPs were assigned to ST179 and ST16 from CC16. Generally, STs from CC16 are considered to be well acclimated to hospital environments. They have previously been reported to cause human infections, acquire exogenous genes via recombination, and be able to carry *van*A or *van*B genes, conjugative plasmids, and transposons involved in the genetic transfer of resistance and virulence in hospital-derived isolates [46–48]. All 18 isolates belonging to CC16 from various specimen types in this study presented MDR phenotypes and genotypes. In addition, they carried virulence genes, most commonly *gel*E and *asa*1, which accords with a previous study [49]. In contrast to previous reports, CC2 isolates were vancomycin sensitive, which supports the hypothesis that this clone was originally vancomycin susceptible and later subsets acquired the vancomycin-resistance gene. In addition, several STs such as ST21, ST480, and ST40 detected in this study were previously reported in China, Tunisia, France, and Spain from human subjects, hospitalized patients, and wastewater, and shared the same characteristics in terms of high-dose antibiotic-resistant phenotypes, resistance genotypes, and virulence genes [50–52]. The lack of information on the population structure of *E. faecalis* in neighboring countries makes it difficult to speculate about the regional spread of these novel STs and other STs detected in Saudi Arabia.

## Conclusion

This study provided the first insights into the population structure of *E. faecalis* isolates from healthcare facilities in the western region of Saudi Arabia. The threat of antimicrobial-resistant *E. faecalis* is steadily increasing, with diverse clonal composition and domination by lineages associated with nosocomial infection in Saudi Arabia. MDR isolates of *E. faecalis* acquired an increased number of virulence gene and diverse patterns of ARGs. Evaluation of antimicrobial susceptibility suggests that ampicillin, tigecycline, and linezolid could be used as treatment options for combatting aminoglycoside- and macrolide-resistant *E. faecalis* strains in healthcare facilities in Saudi Arabia. The appearance of new STs in the studied hospital could be a warning about the emergence and rapid evolution of this clinically important resistant bacteria, and it suggests the necessity of active surveillance in other hospitals and areas of the country.

## Additional file

Additional file 1: **Figure S1.** Percentage distribution of hospital-acquired infection (HA), and community-acquired infection (CA) infections of *E. faecalis* in different disease patients. **Figure S2.** Distribution of the *E. faecalis* isolates analyzed in this study based on nationalities. The number of isolates is mentioned with the country name. **Figure S3.** Heat map of antimicrobial resistance and susceptibility patterns among the 155 *E. faecalis* isolates. **Figure S4.** UPGMA dendrogram of pulsed-field gel electrophoresis (PFGE) bands patterns from the clinical isolates of *E. faecalis*. **Figure S5.** eBURST analysis of the STs from *E. faecalis* isolates. The pink nodes indicate the STs detected in this study that were present in the MLST database. Green nodes indicate the novel STs detected in this study and red circled. **Figure S6.** Distribution of antimicrobial resistance genes among clinical *E. faecalis* isolates. Total 34 ARGs and variants were retrieved from the genomes sequence of the 44 isolates. The y-axis values are expressed in number. **Figure S7.** Distribution of virulence genes retrieved from the genomes sequence of *E. faecalis* isolates. Total 22 virulence-associated genes were identified in this study, and among them, six genes (*cOB*1*, Srt*A*, ebp*B*, ebp*A*, efa*Afs*,* and *ace*) were commonly found in the 44 isolates. The y-axis values are expressed in number. **Table S1.** Distribution of *E. faecalis* isolates according to demographic and clinical data. **Table S2**. Distribution of insertion sequences (ISs) in the *E. faecalis* genomes. (PDF 882 kb)

## Abbreviations

AMR: Antimicrobial resistance
ARG-ANNOT: Antibiotic resistance Gene-ANNOTation
ARGEs: Antimicrobial resistance genes
CARD: Comprehensive antibiotic resistance database
CC: Colonel complex
ECDC: European centre for disease prevention and control
ENA: European Nucleotide Archive
KAUH: King Abdulaziz university hospital
MALDI-TOF: Matrix assisted laser desorption ionization - time of flight
MDR: Multidrug-resistant
MIC: Minimum inhibitory concentration
MLST: Multilocus sequence typing
PFGE: Pulsed-field gel electrophoresis
SNPs: Single nucleotide polymorphisms
ST: Seventeen sequence type
UPGMA: Unweighted pair group with arithmetic mean
UTI: Urinary tract infection
VRE: Vancomycin-resistant enterococci
