# Supplementary material for: Genomic analysis of multidrug-resistant clinical Enterococcus faecalis isolates for antimicrobial resistance genes and virulence factors from the western region of Saudi Arabia
Source: Antimicrob Resist Infect Control. 2019 Mar 25;8:55. doi: 10.1186/s13756-019-0508-4 (PMC6434864; doi:10.1186/s13756-019-0508-4)
Supplement: Supplementary file 1 — Figure S1. Percentage distribution of hospital-acquired infection (HA), and community-acquired infection (CA) infections of E. faecalis in different disease patients. Figure S2. Distribution of the E. faecalis isolates analyzed in this study based on nationalities. The number of isolates is mentioned with the country name. Figure S3. Heat map of antimicrobial resistance and susceptibility patterns among the 155 E. faecalis isolates. Figure S4. UPGMA dendrogram of pulsed-field gel electrophoresis (PFGE) bands patterns from the clinical isolates of E. faecalis. Figure S5. eBURST analysis of the STs from E. faecalis isolates. The pink nodes indicate the STs detected in this study that were present in the MLST database. Green nodes indicate the novel STs detected in this study and red circled. Figure S6. Distribution of antimicrobial resistance genes among clinical E. faecalis isolates. Total 34 ARGs and variants were retrieved from the genomes sequence of the 44 isolates. The y-axis values are expressed in number. Figure S7. Distribution of virulence genes retrieved from the genomes sequence of E. faecalis isolates. Total 22 virulence-associated genes were identified in this study, and among them, six genes (cOB1, SrtA, ebpB, ebpA, efaAfs, and ace) were commonly found in the 44 isolates. The y-axis values are expressed in number. Table S1. Distribution of E. faecalis isolates according to demographic and clinical data. Table S2. Distribution of insertion sequences (ISs) in the E. faecalis genomes. (PDF 882 kb) [file 13756_2019_508_MOESM1_ESM.pdf]

Supplementary Data

Genomic analysis of multidrug-resistant clinical *Enterococcus faecalis* isolates for antimicrobial resistance genes and virulence factors from the western region of Saudi Arabia

Muhammad Farman†[1,2], Muhammad Yasir[1,6]†[*], Rashad R. Al-Hindi[2], Suha A. Farraj[1], Asif A. Jiman-Fatani[3,4], Maha Alawi[3,5], Esam I. Azhar[1,6]

[1]Special Infectious Agents Unit, King Fahd Medical Research Center, King Abdulaziz University, Jeddah, Saudi Arabia

[2]Biology Department, Faculty of Science, King Abdulaziz University, Jeddah 21589, Saudi Arabia

[3]Department of Medical Microbiology and Parasitology, Faculty of Medicine, King Abdulaziz University, Jeddah, Saudi Arabia

[4]Clinical and Molecular Microbiology Laboratories, King Abdulaziz University Hospital, Jeddah, Saudi Arabia

[5]Infection Control & Environmental Health Unit, King Abdulaziz University Hospital, King Abdulaziz University, Jeddah, Saudi Arabia

[6]Department of Medical Laboratory Technology, Faculty of Applied Medical Sciences, King Abdulaziz University, Jeddah, Saudi Arabia

†Authors equally contributed in this study

***Corresponding author:** Muhammad Yasir

Special Infectious Agents Unit, King Fahd Medical Research Center, King Abdulaziz University, Jeddah-21589, Saudi Arabia

Email: yasirkhattak.mrl@gmail.com

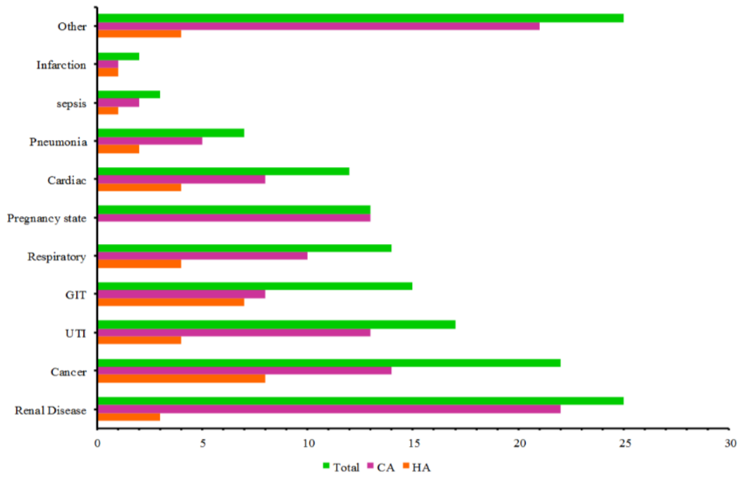

Figure S1. Percentage distribution of hospital-acquired infection (HA), and community-acquired infection (CA) infections of *E. faecalis* in different disease patients.

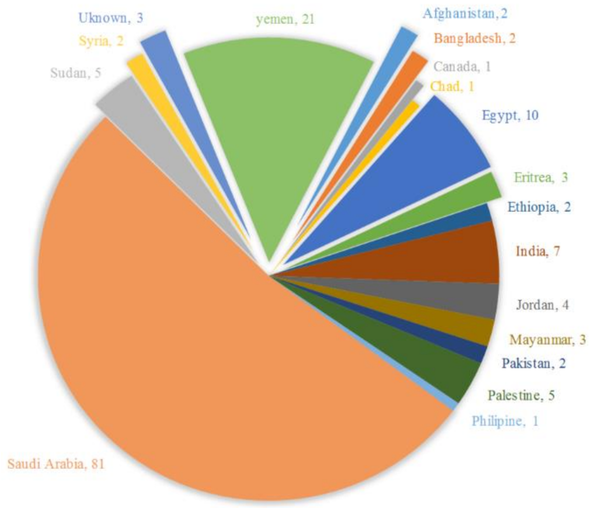

Figure S2. Distribution of the *E. faecalis* isolates analyzed in this study based on nationalities. The number of isolates is mentioned with the country name.

155 *E. faecalis* isolates.

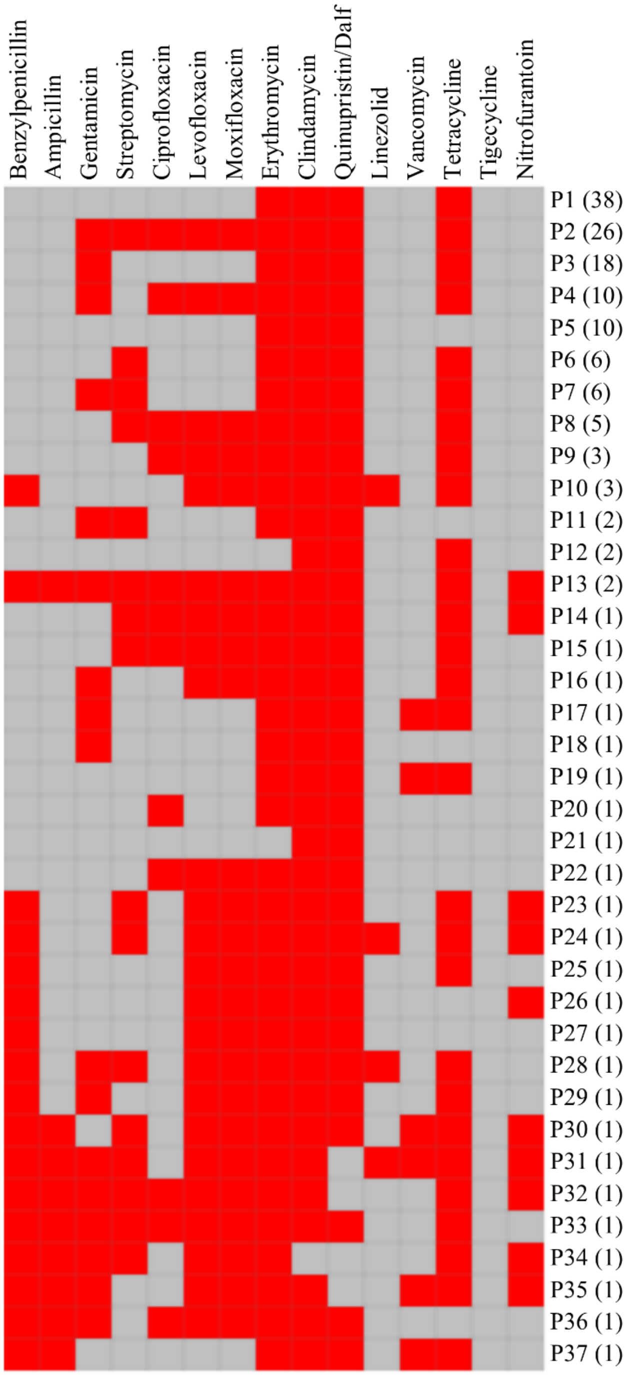

Figure S3. Heat map of antimicrobial resistance and susceptibility patterns among the

155 *E. faecalis* isolates.

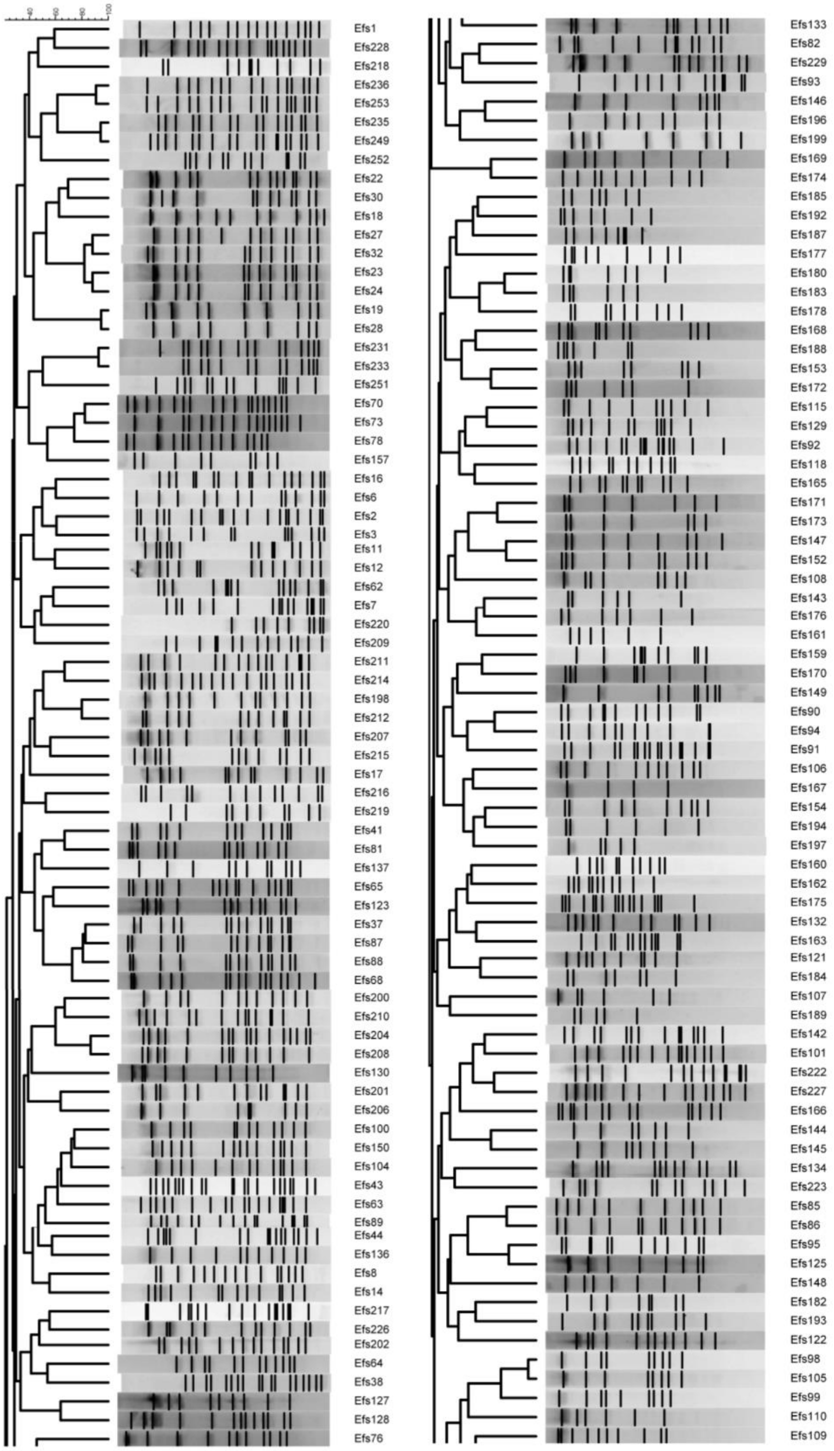

Figure S4. UPGMA dendrogram of pulsed-field gel electrophoresis (PFGE) bands patterns from the clinical isolates of *E. faecalis*.

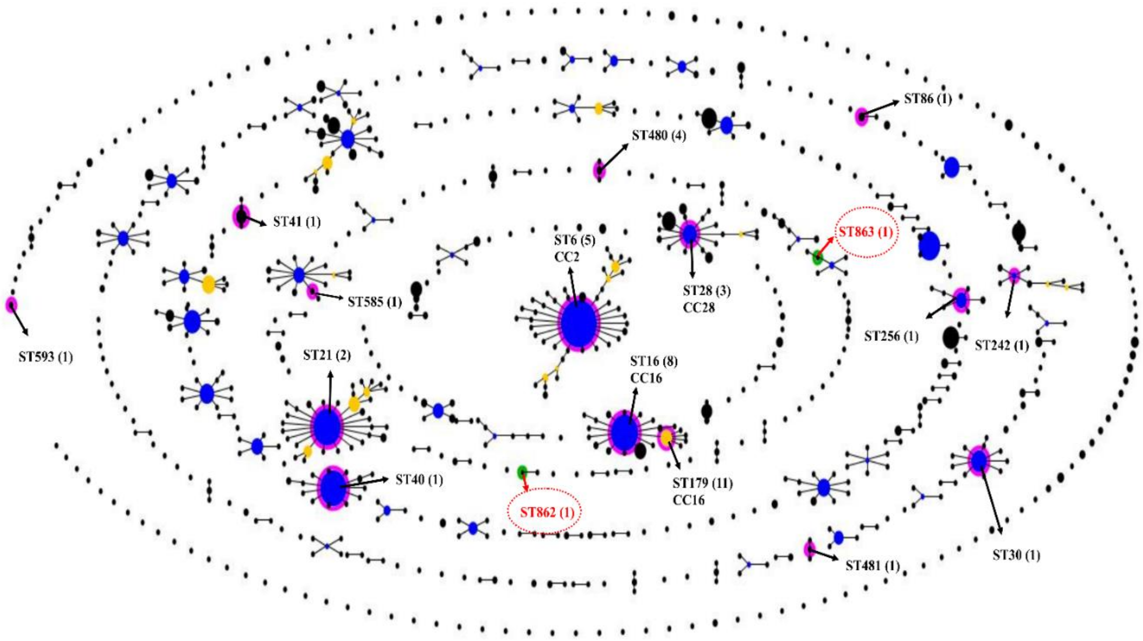

Figure S5. eBURST analysis of the STs from *E. faecalis* isolates. The pink nodes indicate the STs detected in this study that were present in the MLST database. Green nodes indicate the novel STs detected in this study and red circled.

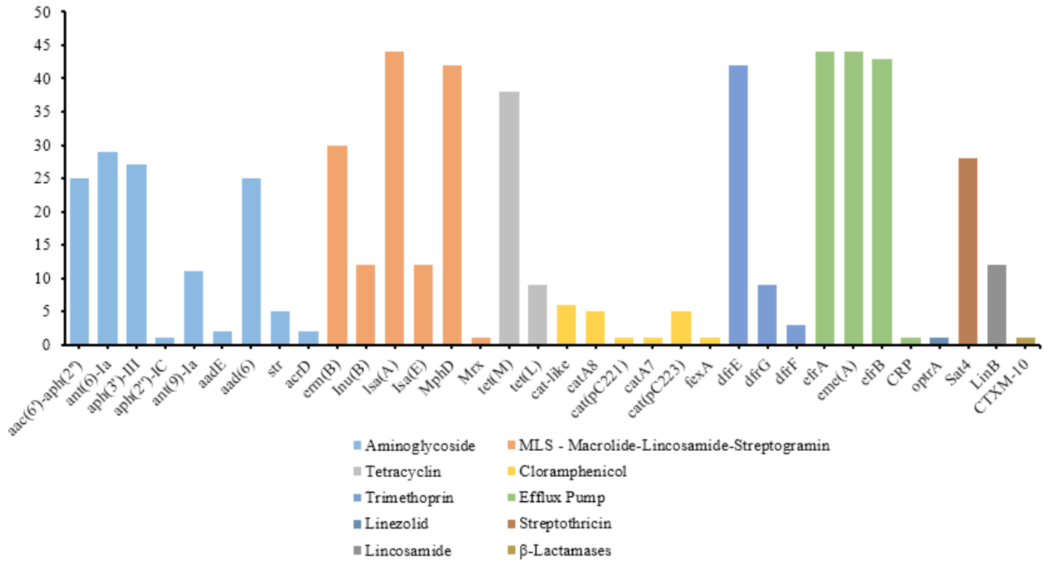

Figure S6. Distribution of antimicrobial resistance genes among clinical *E. faecalis* isolates. Total 34 ARGs and variants were retrieved from the genomes sequence of the 44 isolates. The y-axis values are expressed in number.

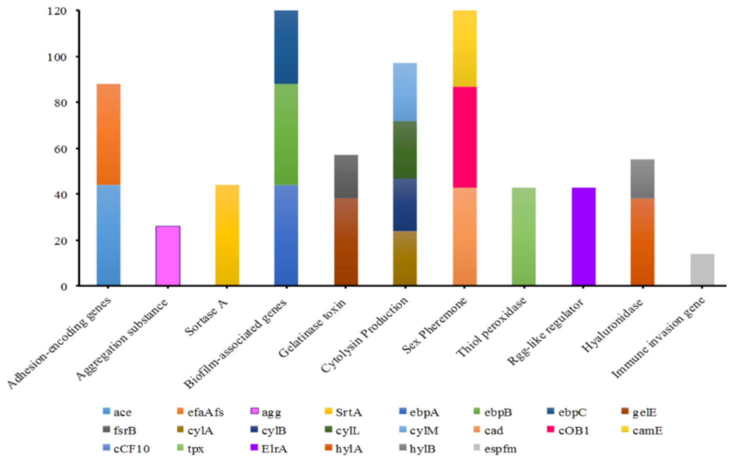

Figure S7. Distribution of virulence genes retrieved from the genomes sequence of *E. faecalis* isolates. Total 22 virulence-associated genes were identified in this study, and among them, six genes (*cOB*1, *Srt*A, *ebp*B, *ebp*A, *efa*Afs, and *ace*) were commonly found in the 44 isolates. The y-axis values are expressed in number.

Table S1. Distribution of *E. faecalis* isolates according to demographic and clinical data.

	Source	Total (155)	Inp n = 102 (65.8%)	Outp n = 53 (34.2%)	P-value	HA n = 38 (24.5%)	CA n = 117 (75.5%)	P-value
**Gender**	Male	83 (53.5)	62 (60.7)	21 (39.6)	0.01	24 (63.2)	59 (50.4)	0.17
	Female	72 (46.4)	40 (39.2)	32 (60.3)		14 (36.9)	58 (49.5)	
**Age**	≤ 1	23 (14.8)	19 (5.8)	4 (7.5)	0.07	7 (18.4)	16 (13,6)	0.61
	2-11	13 (8.3)	8 (7.8)	5 (9.4)		5 (13.5)	21 (17.9)	
	12-18	3 (1.9)	2 (1.9)	1 (1.9)		1 (2.6)	2 (1.8)	
	19-50	49 (31.6)	25 (24.5)	24 (45.2)		9 (23.6)	40 (34.1)	
	>50	67 (43.2)	48 (47)	19 (35.8)		19 (50)	48 (41.1)	
**Nationality**	Saudis	81 (52.2)	48 (47)	33 (62.2)	0.17	17 (44.7)	64 (54.7)	0.04
	Yemini	21 (13.5)	16 (15.6)	5 (9.4)		3 (7.9)	18 (15.3)	
	Egyptian	10 (6.4)	6 (5.8)	4 (7.5)		3 (7.9)	7 (6)	
	Indian	7 (4.5)	7 (6.8)	0		3 (7.9)	4 (3.4)	
	Palestinian	5 (3.2)	3 (2.9)	2 (3.8)		3 (7.9)	2 (1.8)	
	Eritrean	3 (1.9)	2 (1.9)	1 (1.9)		1 (2.6)	2 (1.8)	
	Bangladeshi	2 (1.2)	1 (0.9)	1 (1.9)		2 (5.2)	0	
	Others	26 (16.7)	19 (18.6)	7 (13.2)		6 (15.7)	20 (17.1)	
**Specimens Type**	Urine-midstream	58 (37.4)	35 (34.3)	23 (43.3)	0.03	9 (23.6)	49 (41.8)	0.05
	Urine-catheter	49 (31.6)	35 (34.3)	14 (26.4)		14 (36.9)	35 (29.9)	
	Wound swab	13 (8.3)	5 (4.9)	8 (15.1)		4 (10.5)	9 (7.7)	
	Blood	11 (7.1)	10 (9.8)	1 (1.9)		6 (15.7)	5 (4.3)	
	Others	24 (15.4)	17 (16.6)	7 (13.2)		5 (13.5)	19 (16.2)	

Inp, Inpatient; Outp, Outpatient; HA, Hospital-acquired; CA, Community-acquired.

Table S2. Distribution of insertion sequences (ISs) in the *E. faecalis* genomes.

Isolate Number	STs	Insertion sequences	IS Families
Efs6	ST480	IS*Ef1*	IS*256*
Efs7	ST481	No hits found	-
Efs14	ST21	IS*6770*	IS*30*
Efs16	ST30	IS*6770*	IS*30*
Efs24	ST593	IS*16*	IS*256*
Efs30	ST41	IS*1062*	IS*30*
Efs41	ST21	IS*6770*	IS*30*
Efs44	ST16	IS*Ef1*	IS*256*
Efs62	ST179	IS*6770*	IS*30*
Efs76	ST179	IS*6770*	IS*30*
Efs88	ST480	IS*Ef1*	IS*256*
Efs93	ST480	IS*Ef1*	IS*256*
Efs107	ST179	IS*6770*	IS*30*
Efs110	ST16	IS*Ef1*	IS*256*
Efs122	ST179	IS*6770*	IS*30*
Efs128	ST16	IS*Ef1*	IS*256*
Efs133	ST40	IS*6770*	IS*30*
Efs136	ST179	IS*6770*	IS*30*
Efs142	ST28	IS*Enfa2*	IS*1182*
Efs144	ST86	IS*Enfa364*	IS*30*
Efs145	ST28	IS*Enfa2*	IS*1182*
Efs149	ST179	IS*6770*	IS*30*
Efs153	ST6	IS*6770*	IS*30*
Efs154	ST179	IS*6770*	IS*30*
Efs162	ST862	IS*6770*	IS*30*
Efs163	ST16	IS*Ef1*	IS*256*
Efs165	ST179	IS*6770*	IS*30*
Efs166	ST6	IS*6770*	IS*30*
Efs171	ST28	IS*Enfa2*	IS*1182*
Efs173	ST863	IS*Ef1*	IS*256*
Efs175	ST16	IS*Ef1*	IS*256*
Efs178	ST16	IS*Ef1*	IS*256*
Efs180	ST179	IS*6770*	IS*30*

Efs183	ST585	IS*Ef1*	IS*256*
Efs185	ST16	IS*Ef1*	IS*256*
Efs187	ST179	IS*6770*	IS*30*
Efs189	ST179	IS*6770*	IS*30*
Efs196	ST6	IS*Ef1*	IS*256*
Efs197	ST6	IS*6770*	IS*30*
Efs208	ST480	IS*Ef1*	IS*256*
Efs210	ST16	IS*Ef1*	IS*256*
Efs212	ST6	IS*6770*	IS*30*
Efs215	ST256	IS*1062*	IS*30*
Efs216	ST242	IS*Enfa364*	IS*30*
